# The modulating effects of astaxanthin on apoptosis in women with polycystic ovarian syndrome: A randomized clinical trial 

**DOI:** 10.22038/AJP.2023.23111

**Published:** 2024

**Authors:** Masoome Jabarpour, Ashraf Aleyasin, Maryam Shabani Nashtaei, Mahshad Khodarahmian, Sara Lotfi, Fardin Amidi

**Affiliations:** 1 *Department of Anatomy, School of Medicine, Tehran University of Medical Sciences, Tehran, Iran *; 2 *Department of Infertility, Shariati Hospital, Tehran University of Medical Sciences, Tehran, Iran *; 3 *Department of Infertility, Arash Hospital, Tehran University of Medical Sciences, Tehran, Iran *; 4 *Department of Infertility, Yas Hospital, Tehran University of Medical Sciences, Tehran, Iran *

**Keywords:** Astaxanthin, Polycystic ovary syndrome, Apoptosis, Granulosa cells

## Abstract

**Objective:**

Astaxanthin (ASX) is a lipid-soluble keto-carotenoid with several biological effects. These effects may benefit polycystic ovarian syndrome (PCOS) patients. Imbalanced apoptosis/anti-apoptosis signaling has been considered the major pathogenesis of PCOS. In a randomized clinical trial, we tested the impact of ASX on the apoptotic pathway in PCOS granulosa cells (GCs). The present study hypothesizes that ASX may improve apoptosis in PCOS patients.

**Materials and Methods:**

This trial recruited patients with confirmed PCOS. A total of 58 patients were randomly assigned to take ASX (12 mg) or placebo for 8 weeks. Aspirated follicular fluid (FF) and blood samples were taken from both groups to measure *BAX* and *BCL2* protein expression. Following FF aspiration, GCs from both groups were obtained; Real-Time PCR and Western blotting were used to evaluate the apoptotic pathway’s gene and protein expression levels in GCs.*BAXBCL2*

**Results:**

In GCs analysis, ASX reduced *DR5* gene and protein expression after 8 weeks compared to placebo(p<0.05). Also, *Caspase8 *(p>0.05) and *BAX *(p<0.05) gene expression declined, although the difference was not statistically significant for *Caspase8*. Besides,ASX treatment contributed to an elevated *BCL2* gene expression in GCs(p<0.05). In FF and serum analysis, a statistically significant increase was found in *BCL2* concentration in the ASX group (p<0.05). Moreover, a reduction in *BAX* level was confirmed in both FF and serum of the ASX group; however, this change was not significant in the serum (p>0.05).

**Conclusion:**

It seems that ASX consumption among women with PCOS improved serum and FF levels of apoptotic factors and modulated genes and protein expression of the apoptosis pathway in GCs. Nevertheless, further investigations are needed to reveal the potential role of this compound in PCOS treatment.

## Introduction

Polycystic ovary syndrome (PCOS) is a highly heterogeneous endocrine and metabolic disorder responsible for infertility in approximately 6-10% of women of reproductive age, depending on which diagnostic criteria are applied (McLuskie and Newth, 2017). PCOS has several symptoms, including chronic ovulation disorder, hyperandrogenism, polycystic ovaries, irregular menstrual cycles, hirsutism, acne, and obesity (McLuskie and Newth, 2017; Abraham Gnanadass et al., 2021). PCOS is the most prevalent cause of anovulatory infertility. Anovulatory infertility is characterized by the absence of follicular rupture and the release of an ovum as a result (Brugo-Olmedo et al., 2001). In addition, hormonal dysregulation (e.g. hyperandrogenic state, excessive levels of the anti-Mullerian hormone, insulin resistance, and abnormal gonadotropin levels) leads to anovulation in PCOS women that may result in infertility (Abraham Gnanadass et al., 2021). While the pathogenesis of PCOS is not completely understood, research has shown that this disease is linked to oxidative stress (OS) (Zuo et al., 2016). A situation of OS occurs when a balance is not achieved between producing and scavenging reactive oxygen/nitrogen species (ROS/RNS) (Pisoschi and Pop, 2015). Overproduction of ROS can induce mitochondria-mediated apoptosis (Hyatt et al., 2019).In addition to the role of ROS in cell signaling, ROS is also crucial in regulating the primary pathways that lead to apoptosis. These pathways are controlled by mitochondria, death receptors, and the endoplasmic reticulum (ER) (Redza-Dutordoir and Averill-Bates, 2016). Studies have revealed that excessive accumulation of ROS is connected with ER stress, which might further accelerate cell death (Fujii et al., 2018; Ozgur et al., 2018). Overexpression of the UPR transcription factor C/EBP homologous protein (CHOP) and upregulation of other UPR genes are engaged in cell death induced by ER stress (Urra et al., 2013). It has been shown that the death receptor 5 (*DR5*), a CHOP target, is vital in inducing apoptosis by ER stress by boosting the synthesis of the autocrine death-ligand signal. This phenomenon is independent of its external ligand, i.e. TNF-related apoptosis-inducing ligand (TRAIL) (Lu et al., 2014; Yamaguchi and Wang, 2004; Zlotorynski, 2014). Furthermore, it has been demonstrated that ROS (Trivedi et al., 2014; Chang et al., 2015) is associated with an increase in *DR5* expression. Moreover, ER stress in PCOS induces apoptosis of GCs in antral follicles by activating the *DR5* expression (Azhary et al., 2019). OS, apoptosis caused by OS, and mitochondrial dysfunction have been detected in the GCs of PCOS patients (Hyderali and Mala, 2015; Fan et al., 2012; Ávila et al., 2016). GCs apoptosis has also been demonstrated in PCOS animal models and patients (Salehi et al., 2017; Zhao et al., 2013; Ding et al., 2016). PCOS is commonly associated with abnormal folliculogenesis, including low rates of follicular atresia and incomplete maturation of antral follicles (Zehravi et al., 2021; Seifer and Merhi, 2014). Apoptosis imbalance within GCs has been linked to disruption of folliculogenesis, as GCs are absolutely essential to the maturation of oocytes (Asselin et al., 2000; Das et al., 2008). In addition to the maturation of the oocyte, GCs are crucial to fertilization and implantation (Lai et al., 2017; Lai et al., 2018). Hence, GCs apoptosis is linked to poor in vitro fertilization (IVF) success rates and oocyte quality in PCOS patients (Ávila et al., 2016; Lai et al., 2018). A remaining challenge in this regard is to develop an effective treatment for patients with PCOS. In addition to conventional therapies, herbal medicine can be used to treat PCOS patients. PCOS is characterized by a significant reduction in antioxidants and increased oxidative stress risk. It has been suggested that antioxidant supplementation may improve PCOS management (Gharaei et al., 2021).

Astaxanthin (ASX) is a red keto-carotenoid pigment. Previous studies have shown that ASX benefits healthregarding its biological origin and high antioxidant potency. These health benefits include anti-tumor, anti-inflammatory, anti-apoptotic, hepatoprotective, heart protection, and immunomodulatory effects (Ikeuchi et al., 2007; Pashkow et al., 2008; Hussein et al., 2006). Several studies have focused on the potential effects of ASX on the reproductive system of mammals. Research has shown that ASX supplementation could improve the maturation and quality of oocytes, enhance embryo development, and exert protective effects on the endometrium, oviduct, and placental trophoblast cells in the female reproductive system (Abdel-Ghani et al., 2019; Ispada et al., 2018; Xiang et al., 2021; Lord and Aitken, 2013; Jang et al., 2010; Wan et al., 2020). The randomized controlled trial study published by our laboratory revealed that ASX activated the Nrf2 signaling pathway to protect PCOS women from OS (Gharaei et al., 2022). We reported similar results regarding ASX in *ex vivo* human granulosa cells (HGCs) (Eslami et al., 2021). Additionally, this substance has been found effective in animal models of PCOS (Ebrahimi et al., 2021). In light of the evidence of anti-apoptotic effects of ASX in various studies (Dong et al., 2013; Guo et al., 2015; Zhang et al., 2014), this trial aimed to assess the effects of ASX supplementation on the apoptosis pathway in GCs of infertile PCOS patients.

## Materials and Methods

**Study design**

This triple-blind randomized clinical trial was conducted at the Omid fertility clinic, Tehran, Iran, between November 2020 and September 2021. The study included 58 infertile women aged 18 to 40 years diagnosed with PCOS based on Rotterdam criteria (ESHRE and Group, 2004) who were candidates for IVF. Women with PCOS were allocated to receive ASX supplement (n=29) or placebo (n=29) in a 1:1 ratio for a period of 8weeks. Tehran University of Medical Sciences (TUMS) research ethics committee approved this study in terms of adherence to the Declaration of Helsinki.All subjects signed informed consent forms before enrollment (ethics committee reference number: IR.TUMS.REC.1399.340). Iranian website for clinical trials registration (http://www.irct.ir) was used to register the study (IRCT-ID: IRCT20201029049183N1). In this study, only a part of the results of the clinical trial registered with the IRCT is presented. 

**Participants**

Infertile PCOS patients between the ages of 18 and 40 years who met the Rotterdam criteria were included in this RCT (ESHRE and Group, 2004). PCOS was the only endocrine disorder found in all patients, and they were all advised to undergo intracytoplasmic sperm injection (ICSI). This study excluded patients who had the following conditions:

Severe endometriosis (stages 3 and 4 according to the revised AFS-rAFS classification of endometriosis), follicular stimulating hormone (FSH) >10 mg/ml, hyperprolactinemia, Cushing’s disease, ovarian tumors, thyroid disease, severe male factor infertility (especially non-obstructive azoospermia), drug history affecting ovarian function in the three months before the study (steroids and oral contraceptive pill (OCP), female infertility factors other than cervical and tubal factors, any autoimmune disease, systemic disorders like metabolic syndrome, severe obesity or malnutrition (BMI>35), hyperlipidemia, diabetes, or cardiovascular disease.

**Intervention**

For 8 weeks, participants in the treatment (ASX) group received ASX 12 mg/day (2x6 mg capsules) orally (Astareal, Tokyo, Japan). As a placebo, patients were given 2 capsules per day that were identical to ASX in terms of shape, color, size, taste, and scent. A similar manufacturer made the capsules at the same time. As shown by previous studies, the daily dosage of ASX was set at 12 mg orally (Brendler and Williamson, 2019). It was important that patients inform the researchers of any changes they made to their daily routine or diet. During the experiment, all patients received daily reminders to consume their supplements to maintain compliance.

**Randomization and blinding**

**Figure 1:**
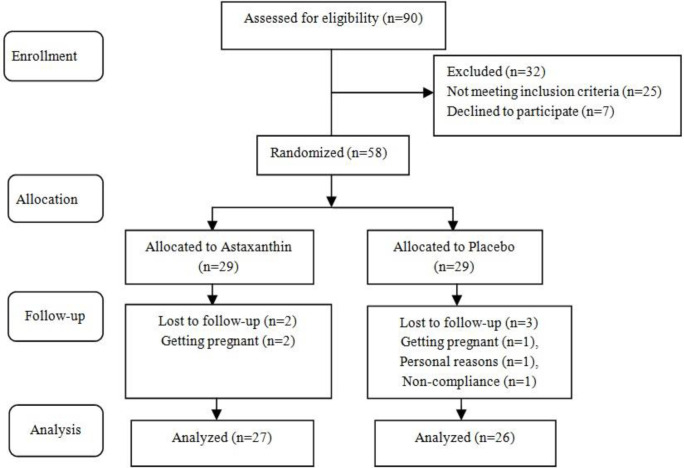
Summary of patient flow through the study

The study began with 58 women. Block randomization was used to allocate the participants to either an intervention (ASX) or a control group (placebo). Figure 1 depicts the plan of the research. Researchers, patients, and the study’s statistician were all unaware of the study’s categories, and the decipherer was outside the research team. The trial participants were randomly given the ASX and placebo capsules in identical bottles containing 60 capsules. The pharmaceutical content of each bottle was labeled as a code on the bottles by someone outside the research project. Also, the research team was unaware of its interpretation.

**Study outcomes**

Genes and proteins expression levels of the apoptotic pathway were the primary outcomes of this study. This trial’s secondary outcomes were apoptotic indicators in FF and serum.

**Ovarian stimulation protocol**

Patients underwent controlled ovarian stimulation using a flexible antagonistic regimen. Before starting the ovarian stimulation cycle, all patients were prescribed oral contraception (OCP-Ovocept LD®, Abureihan, Iran) for 21 days. As a brief overview, females from the third day of their period were prescribed 150-300 IU/day of recombinant follicle-stimulating hormone (r-FSH; Gonal-F, Merck Serono SA, Switzerland). The most appropriate dosage was determined based on estradiol concentration and the ovarian response. By monitoring the ovaries, when two or more follicles with the size of 14-15 mm were detected, they were administered with 0.25 mg/day of cetrotide (Merck Serono SA, Switzerland) and an antagonist of gonadotropin-releasing hormone (GnRH). Cetrotide was stopped once ≥2 follicles reached 18 mm in diameter, and 0.2 mg triptorelin acetate (Decapeptyl; Ferring Gmbh, Germany) with 2,000 IU HCG (Ovitrelle, Merck Serono SA, Switzerland) was given instead (Ovitrelle, Merck Serono SA, Switzerland). The oocytes were aspirated via transvaginal ultrasound after 36 hr (Qasemi et al., 2021).

**Sample preparation**

At the start and end of the trial, 12 ml of venous blood was collected from participants following a 12-hr overnight fasting. Centrifugation was used to separate serum from samples; sera were then stored at -80°C until analysis. GCs were isolated as previously described (Qasemi et al., 2021). On the day of oocyte retrieval, follicles were aspirated without blood contamination. Following oocyte retrieval, FF (including GCs and heterogeneous cells) was centrifuged for 15 min at 3000g. About 5 ml of the supernatant (i.e. FF) was aliquoted and stored at -80°C to determine apoptotic factors. GCs were separated by density gradient centrifugation of the FF-derived pellet over 5 ml of Ficoll-Hypaque (Lymphodex, Inno-Train, Germany). After centrifuging for 20 min at 400 g, cells from the interface were collected and considered for analysis.

**Real-time quantitative reverse transcription-polymerase chain reaction (RT-PCR)**

RT-PCR was accomplished using RNX-PLUS^TM^ (Sinnacolon, Tehran, Iran) to obtain total RNA, the cDNA synthesis kit (Sinnacolon, Tehran, Iran) to create cDNA, and the Real Q Plus 2x Master Mix Green (Amplicon, Denmark) for qRT-PCR, all as directed by the manufacturer. In a reaction mix with a final volume of 25 μl, the following components were added: 0.5 μl of forward primer, 0.5 μl of reverse primer, 12.5 μl of RealQ plus 2x Master Mix, 0.5 μl of cDNA template, and 11 μl of double-distilled (DD) water. All genes had the following PCR cycling parameters 15 min at 95°C followed by 40 cycles of 95°C for 15 sec, 60°C for 30 sec, and 72°C for 30 sec. Table 1 displays the primers utilized in this study. The mean value of genes were determined by doing all the examinationsin duplicate.Glyceraldehyde‐3‐phosphate dehydrogenase (*GAPDH*) was used as a control housekeeping gene.All transcripts were measured by Real-Time PCR System (Applied Biosystems, Darmstadt, Germany).In addition, the Livak method (2 ^-ΔΔCt^) was used to determine the relative expression levels of genes (Livak and Schmittgen, 2001).

**Table 1:** Specific primers used for real-time quantitative PCR

**Gene**	**Primer**	**Product** **length**
*DR5*	F: AGCACTCACTGGAATGACCTCC R: GTGCCTTCTTCGCACTGACACA	119
*BAX*	F: TCAGGATGCGTCCACCAAGAAG R: TGTGTCCACGGCGGCAATCATC	103
*BCL2*	F: GATGGGATCGTTGCCTTATGC R: CAGTCTACTTCCTCTGTGATGTTGT	105
*CASPASE 8*	F: AGAAGAGGGTCATCCTGGGAGA R: TCAGGACTTCCTTCAAGGCTGC	142
*GAPDH*	F: CGCCAGCCGAGCCACATC R: CGC CCA ATA CGA CCA AAT CCG	162

*DR5* death receptor 5, *BAX* Bcl-2 Associated X-protein, *BCL2* B-cell lymphoma 2, *CASPASE8* cysteine-aspartic acid protease8, GAPDH glyceraldehyde-3-Phosphate dehydrogenase

**Western blot**

The Western blot was performed as described previously (Jabarpour et al., 2018). The total protein was extracted by lysing the cells in Ripa buffer. Protein concentrations were determined using the Bradford assay. Afterward, proteins were separated by SDS/PAGE and transferred to a polyvinylidene difluoride (PVDF) membrane. After blocking with 5% milk for 1 hr, proteins were incubated for 1 hr at room temperature with primary antibodies (anti- *DR5*(Cat No: ab199357, Abcam) and anti-beta actin-loading control (Cat No: ab8227; Abcam)). Membranes were washed several times and incubated for 1 hr at room temperature with secondary antibodies (goat anti-rabbit IgG H&L (HRP); Cat No: ab6721; Abcam). The immunoreactive bands were visualized by incubating the membranes for 1-2 min with enhanced chemiluminescence (ECL). The membranes were scanned using an HP Scan Jet G3110 apparatus (Hewlett Packard Company CA, USA). Moreover, β-actin was used to normalize protein expression. The gel analyzer Version 2010a software (NIH, USA) was used to perform densitometry on protein bands. This process was determined by dividing the percentage area under the curve of each band by the percentage area under the curve of its corresponding actin band. Eventually, the resulting computed values were compared among groups.

**Enzyme-linked immunosorbent assay (ELISA)**

Enzyme-linked immunosorbent assay (ELISA) concentrations of *BAX* (Cat No: LS-F26697) and *BCL2* (Cat No: LS-F10922) were determined according to the manufacturer’s protocol in FF and serum (before and after the intervention). The assay sensitivity for *BAX* and *BCL2* was less than 1 pg/ml and less than78 pg/ml, respectively. The inter- and intra-assay coefficients of variation were, respectively, <10% and <9% for *BAX* and <6.3% and <3.8% for *BCL2*.

**Statistical analysis**

The normality of parameters was examined using the Kolmogorov-Smirnov test. The data are expressed as means±SD (standard deviation). Due to the lack of comparable research on primary outcomes, Cohen’s effect size was applied to determine the sample size. With a Cohen effect size of 0.8, a 10% drop-out rate, 80% study power, and 5% type I error, each group included 29 participants. In the statistical analysis, the independent sample t-test or paired student’s t-test was performed with SPSS version 22 and at a significance level of p<0.5. 

## Results

**Comparison of baseline parameters between the groups**

In this study, 5 patients were excluded during the intervention phase, including 3 participants in the placebo group and 2 in the ASX group. Finally, the total number of patients reached 53 (27 for the intervention and 26 for the placebo) (Figure 1). 

**Table 2:** Baseline parameters of individuals in the two study groups

		**Mean±SDPlacebo(n=26)**	**Mean±SD ASX (n=27)**	**p**
**Age(years)**		30.84±4.84	30.36±5.16	0.745
**BMI(kg/m2)**	**baseline**	26.24±1.59 26.20±1.59 0.03±0.16	26.12±1.56 26.09±1.48 -0.03±0.5	0.802 0.802 0.977
	**end**			
	**change**			
**Infertility duration(year)**		3.386±1.93	4.24±2.02	0.147
**Mean menstruationduration(day)**		6.81±0.9	6.48±1.29	0.338
**Mean menstrual cycleduration(day)**		42.36±10.05	44.12±14.4	0.634
**Smoking**	**Yes**	1	0	0.303^a^
	**No**	25	27	
**Alcohol Consumption**	**Yes**	2	1	0.610^a^
	**No**	24	26	
**Baseline FSH (μIU/ml)**		3.92±1.11	4.19±1.15	0.416
**Baseline LH (μIU/ml)**		9.01±3.54	8.85±3.13	0.874
**Baseline Tes (ng/ml)**		1.18±0.56	1.24±0.55	0.713
**Baseline AMH(ng/ml)**		9.21±1.96	8.06±3.01	0.132
**Baseline PRL (ng/ml)**		12.36±1.8	13.14±2.27	0.203

Data based on mean±SD (independent t-test). ^a ^Based-on Chi-Square test. *BMI* body mass index, *FSH* follicle-stimulating hormone*, LHluteinizing hormone, Tes* testosterone*, AMH *anti-Müllerian hormone*, PRL. *prolactin

During the trial, no one in the study experienced any side effects due to the ASX supplementation, and participants complied with the intervention well. As of the beginning of the trial, there were no statistically significant differences between the control and ASX groups (Table 2) in terms of infertility length, mean age, BMI, and hormonal profile. 

**Effects of treatments on mRNA levels of** ***DR5, BAX, BCL2, and CASPASE*** **8**

**Figure 2:**
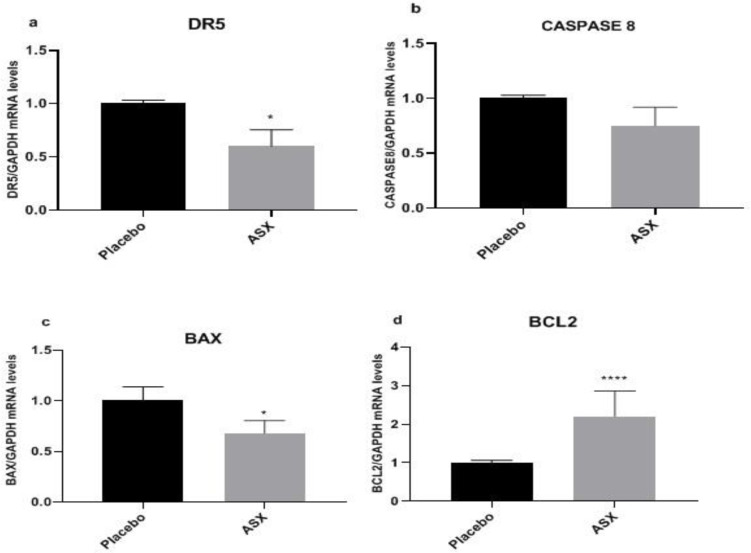
The fold changes levels of *DR5* (a), *CASPASE8* (b), *BAX* (c), and *BCL2* (d) in GCs of placebo and ASX groups. Statistical significance (p<0.05) was assessed by t-test. After the intervention, it was found that in the ASX group, the expression levels of *DR5* and *BAX* were significantly decreased compared to the control group(p<0.05), while the reduction level of *CASPASE8* was not significantly different between the two groups (p>0.05). Also, the results showed that the fold change in the level of *BCL2* was significantly increased in the ASX group (p<0.05). Differences between groups; *: p<0.05 and ****: p<0.0001.

According to the gene expression data in GCs, the *DR5*, *Caspase8*, and *BAX* expression levels were lower in the ASX group compared to the placebo group (p<0.012, Figure 2a; p=0.13, Figure 2b; p=0.013, Figure 2c, respectively). While the mRNA expression levels of *DR5* and *BAX* considerably decreased in the ASX group, this reduction in *Caspase8* was not statistically significant. Besides, a considerable increase in *BCL2* expression (p<0.0001, Figure 2d) was observed after ASX administration compared to placebo.

**Effects of treatments on the protein level of DR5**

**Figure 3:**
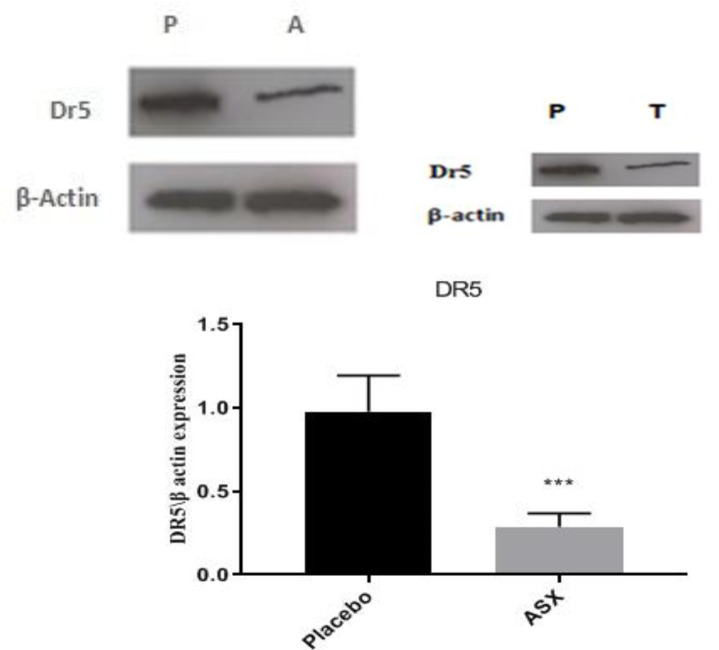
The protein expression level of *DR5* in the GCs of placebo ASX groups. Western blot analyzed the protein expression of *DR5* normalized to β-actin. Following the intervention, the protein expression of *DR5* was significantly reduced in the ASX group compared to the placebo group. Statistical significance (p<0.05) was assessed by t-test. P: placebo; A: ASX. ***: p<0.001

According to the protein expression data in GCs, the *DR5* expression level declined considerably in the ASX group compared to the placebo group (p<0.001, Figure 3).

**Table 3:** FF analysis of *BAX* and *BCL2* concentration

**Variables **	**Mean±SD** **Placebo (n=27)**	**Mean±SD** **ASX (n=28)**	**p**
*BAX*(pg/ml)	125.7±20.45	113.5±18.45	0.037*
*BCL2* (pg/ml)	187.1±29.81	218.8±35.28	0.001*

^P ^Based on independent t-test.*: p<0.05, statistically significant. FF follicular fluid, *BAX* Bcl-2-associated X protein, BCL*2 B*-cell lymphoma 2

**Effects of treatments on serum and FF levels of BAX and BCL2**

Based on the FF results, the *BCL2* (p<0.05) and *BAX* levels (p<0.05) were statistically higher and lower, respectively, in the ASX group (Table 3). Additionally, in the serum analysis, *BCL2* levels rose significantly (p<0.05), but there was no significant change in *BAX* levels between the ASX and placebo groups (p>0.05; Table 4).

**Table 4:** Serum analysis of *BAX* and *BCL2* concentration

**Variables **		**Mean±SD** **Placebo (n=27)**	**Mean±SD ASX (n=28)**	**p**
*BAX*(pg/ml)	Baseline End Mean changes	53.27±12.31 52.32±12.75 -0.95±1.79	54.73±15.41 51.78 ±14.90 -2.63±4.18	0.724 0.894 0.087
*BCL2* (pg/ml)	Baseline End Mean changes	88.55±17.99 90.98±17.74 2.42±9.37	85.76±22.39 94.06±27.05 8.3±8.31	0.642 0.652 0.027*

^P^Based on independent t-test). *: p<0.05, statistically significant. *BAX* Bcl-2-associated X protein, BCL*2 B*-cell lymphoma 2.

## Discussion

Three distinct signaling pathways can initiate apoptosis before caspase activation, including an extrinsic pathway that is mediated by death receptors existing on the surface of the cell (Green and Llambi, 2015) or the intrinsic pathway through mitochondria or the ER (Ferri and Kroemer, 2001; Jin and El-Deiry, 2005). 

Apoptosis via the death receptor is induced by recruiting adaptor proteins Fas-associatedprotein with death domain (FADD) and procaspase-8 or -10 to the receptor’s cytoplasmic surface, where they form thedeath-inducing signaling complex (DISC). Also, *Caspase8/10 *can degrade Bid to produce tBid which activates the mitochondrial apoptotic crosstalk route with death receptors. In this regard,tBid suppresses the anti-apoptotic function of Bcl-2 and Bcl-XL in the mitochondria while promoting the activation of *BAX* and Bak. The mitochondrial apoptosis pathway is triggered due to the release of cytochrome c and Smac/Diablo (Redza-Dutordoir and Averill-Bates, 2016). 

In this clinical trial, we evaluated the effect of ASX on apoptotic factors in GCs of infertile patients with PCOS. In GCs, we identified that administration of ASX leads to a significant reduction in gene and protein expression of *DR5* (p<0.012 and p<0.001, respectively). Afterward, gene expression levels of *CASPASE8* (p=0.13) and *BAX* (p=0.013) decreased; however, this reduction in *Caspase8* was not statistically significant. Besides, ASX treatment contributed to an increase in *BCL2* expression (p<0.0001). In FF and serum analysis, a statistically significant increase was found in *BCL2* concentration in the ASX group (p<0.05). Moreover, a reduction in *BAX* level was confirmed in both FF (p<0.05) and serum of the ASX group, although this change was not statistically significant in the serum (p>0.05). 

Here, experimental evidence presented that ASX could be potent in preventing apoptosis in PCOS infertile women. In this context, mounting data establishes a clear link between ASX’s therapeutic effects and its anti-apoptotic capabilities (Fakhri et al., 2019). Several mechanisms may mediate the anti-apoptotic effects of ASX. ASX inhibits apoptosis by reducing the levels of p-ERK/ERK, cytochrome c, *caspases *3 and 9, and the *BAX*/*BCL2* ratio (Fakhri et al., 2019). For instance, Wang et al. hypothesized that ASX inhibited apoptosis via the PI3K/Akt pathway in isoflurane-treated rat and mouse hippocampus cells to mitigate the deleterious effects of isoflurane (Wang et al., 2016). They found that ASX decreased isoflurane-induced caspase-3 activity both *in vivo* and *in vitro*. ASX also preserves mitochondrial integrity by modulating the p38 and MEK signaling pathways and lowered caspase 3/9, thereby reducing cytochrome c release and inhibiting caspase-dependent apoptotic cell death in human neuroblastoma SH-SY5Y cells exposed to 6-hydroxydopamine (Ikeda et al., 2008). Another study found that ASX appeared to protect against L-glutamate-induced PC12 cell death, mostly via the Bcl-2/*BAX* signalling pathway (Zhang et al., 2015). Cell survival signaling pathways such as UPR and Nrf2 are activated at low levels of ROS. In comparison, cell death signaling pathways such as apoptosis and necroptosis are activated at high levels of ROS. ROS triggers apoptosis by activating pathways in the mitochondria, death receptors, and ER (Redza-Dutordoir and Averill-Bates, 2016). Liu et al. evaluated the effects of ASX and its mechanisms involved in ROS-induced apoptosis using dopaminergic SH-SY5Y cells. They showed that ASX reduces ROS, cytochrome C release, mitochondrial membrane potential, and apoptosis (Liu et al., 2009). Accordingly, apoptosis caused by ROS could be a potential target for ASX to halt the course of various organ damages. Generally, these findings are consistent with our results. The present study showed that ASX had an anti-apoptotic effect by reducing the expression of *BAX* and CASPASE 8and enhancing *BCL2* as an anti-apoptotic factor in GCs. ASX has been found to have anti-apoptotic properties in numerous studies. For instance, Li et al. found that ASX might mitigate the effect of H_2_O_2_ on ARPE-19 cells, thereby preventing apoptosis by inhibiting the expression of the cleaved caspase-3 protein (Li et al., 2013). In addition, ASX has been shown to reduce the induction of apoptosis by H_2_O_2_ in the RGC-5 cell line of the diabetic rat retinal ganglion by decreasing the apoptotic rate of cells as demonstrated by annexin V-FITC/PI staining. They also showed that diabetic *db/db* mice treated with ASX had a lower expression level of the proteins Bcl-2, BAD, and caspase-3. This result may suggest application of ASX in the treatment of diabetic retinopathy in the future (Dong et al., 2013). ASX was found to decrease *BAX* and Caspase-3 expression while increasing Bcl-2 expression in a rat model of spinal cord injury (Masoudi et al., 2017). Since oxidative stress induces apoptosis by raising caspase-3 activity and *BAX*as the pro-apoptotic protein and decreasing the anti-apoptotic protein Bcl-2, ASX reduces apoptosis in various forms by reducing reactive oxygen species (ROS) (Kim and Kim, 2018). Consistent with this finding, it was demonstrated that ASX reduced oxidative stress-induced apoptosis in lung epithelial cells (Song et al., 2014), heart (Fan et al., 2017), and neuroblastoma cells (Ikeda et al., 2008). ASX administration ameliorates coronary microembolization-induced cardiac dysfunction by lowering oxidative stress and apoptosis, activating the Nrf2/HO-1 signaling pathway, and modulating the expression of *BAX* and *BCL2* in rat cardiomyocytes (Xue et al., 2019). Recently, we examined how ASX consumption affects the outcomes of assisted reproductive technology (ART) and oxidative stress in PCOS patients. Our results indicated that, in PCOS patients, therapy with ASX elevated serum TAC levels and activated the Nrf2 axis in GCs (Gharaei et al., 2022). The present work revealed that ASX might have a role in apoptosis protection by activating the Nrf2 axis. Besides, we previously evaluated the effect of ASX on oxidative stress in GCs of the PCOS mouse model. The results showed that ASX was associated with a significant reduction in oxidative stress and, subsequently, apoptosis non-significantly (Ebrahimi et al., 2021). This small discrepancy between this study and our previous study could be explained by differences in the subject species used, the dose of ASX provided, and the duration of ASX exposure. Other research showed that ASX attenuated the effects of busulfan on spermatogonial stem cells by modulating NRF-2/HO-1 and mitochondria-mediated apoptotic pathways (Afzali et al., 2021). Cui et al. discovered that in mice with myocardial injury, ASX increases the expression of NRF-2, HO-1, and Bcl-2 protein and decreases the expression of *BAX*, *Caspase *3, and *Caspase *9 in cardiomyocyte (Cui et al., 2020). In our study, treatment with ASX led to a reduction in *DR5* gene and protein expression level. According to former studies, the expression of *DR5* is promoted by ER stress (Lu et al., 2014; Yamaguchi and Wang, 2004; Zlotorynski, 2014; Azhary et al., 2019) and ROS (Trivedi et al., 2014; Chang et al., 2015) enhancement. Given the evidence that ASX is related to a reduction in ROS (Kim and Kim, 2018) and ER stress (Wang et al., 2021), it is reasonable to assume that ASX may alter *DR5* expression by lowering ROS and alleviating the ER stress pathway. In contrast to our findings that ASX has an anti-apoptotic effect, it has been shown that ASX had a pro-apoptotic effect in K562 cells, thereby supporting its anti-cancer properties. ASX has been proven to inhibit the growth of several cancer cell lines, including liver, breast, and lung cancer cells (Zhang et al., 2011; Kim et al., 2020; Wu et al., 2016; Song et al., 2011). In a hamster oral cancer model, Kavitha et al. discovered that ASX suppresses the ERK/MAPK and PI3K/AKT cascades by activating the intrinsic apoptotic pathway (Kavitha et al., 2013). In another study, Hormozi et al. evaluated the impact of ASX on the LS-180 cell line and found that ASX induced both apoptosis and antioxidant activity. These authors discovered that ASX induced apoptosis by increasing *BAX* and Caspase3 gene expression while decreasing *BCL2* gene expression (Hormozi et al., 2019). Accordingly, it is inferred that ASX may have either anti- or pro-apoptotic effects, based on the clinical situation.

According to our findings, ASX, a natural supplement with anti-apoptotic and antioxidant properties, can influence apoptosis in GCs by modulating the expression of apoptotic genes and proteins.It seems that ASX may improve PCOS by modifying the apoptosis pathway. However, further investigations are needed to reveal the potential role of this compound in PCOS treatment 

## Conflicts of interest

The authors have declared that there is no conflict of interest.
